# A scalable analytical approach from bacterial genomes to epidemiology

**DOI:** 10.1098/rstb.2021.0246

**Published:** 2022-10-10

**Authors:** Xavier Didelot, Julian Parkhill

**Affiliations:** ^1^ School of Life Sciences and Department of Statistics, University of Warwick, Coventry CV4 7AL, UK; ^2^ Department of Veterinary Medicine, University of Cambridge, Cambridge CB3 0ES, UK

**Keywords:** bacterial genomics, infectious disease epidemiology, recombination, dated phylogeny

## Abstract

Recent years have seen a remarkable increase in the practicality of sequencing whole genomes from large numbers of bacterial isolates. The availability of this data has huge potential to deliver new insights into the evolution and epidemiology of bacterial pathogens, but the scalability of the analytical methodology has been lagging behind that of the sequencing technology. Here we present a step-by-step approach for such large-scale genomic epidemiology analyses, from bacterial genomes to epidemiological interpretations. A central component of this approach is the dated phylogeny, which is a phylogenetic tree with branch lengths measured in units of time. The construction of dated phylogenies from bacterial genomic data needs to account for the disruptive effect of recombination on phylogenetic relationships, and we describe how this can be achieved. Dated phylogenies can then be used to perform fine-scale or large-scale epidemiological analyses, depending on the proportion of cases for which genomes are available. A key feature of this approach is computational scalability and in particular the ability to process hundreds or thousands of genomes within a matter of hours. This is a clear advantage of the step-by-step approach described here. We discuss other advantages and disadvantages of the approach, as well as potential improvements and avenues for future research.

This article is part of a discussion meeting issue ‘Genomic population structures of microbial pathogens’.

## 1. Introduction

Over the past decade, the cost and time required to sequence whole bacterial genomes have reduced dramatically [1]. Sequencing is frequently applied to many or all isolates in local outbreaks, or to a high proportion of cases in more endemic situations, as well as large retrospective and longitudinal collections. This genomic data has huge potential to deliver new insights into the evolution and epidemiology of bacterial pathogens, which can lead to better control measures. However, the lack of scalable methodology for analysis of this genomic data represents an important bottleneck for the realization of their full potential.

A gold standard for the analysis of pathogen genomic data has been set by the integrated phylogenetic frameworks implemented for example in BEAST [2] and BEAST2 [3]. These phylodynamic tools were originally conceived for viral genetics and are still mostly used for that purpose, but have also been increasingly applied to bacterial genomic data [4]. One of the strengths of these tools is that they can infer a dated phylogeny by combining the genomic data with the dates of isolation, resulting in estimates for the dates of the common ancestors in the phylogeny. Such dated phylogenies are extremely useful to draw epidemiological interpretations from the genomic data, as we will see. Another advantage of the integrated phylogenetic frameworks is that they include a number of powerful extensions, for example, to use relaxed clock models [5], to estimate past population dynamics [6], geographical spread [7–9] or transmission between hosts [10,11]. This integrated approach has many natural advantages but also limitations especially in terms of scalability to analyse larger datasets.

These limitations of the integrated approach are especially important in bacterial genomics, where the genomes are orders of magnitude longer than in viral genetics and often subject to recombination. The ClonalOrigin model [12] of bacterial evolution has been integrated into BEAST2 [13], but the resulting algorithm is too computationally intense to be applied to whole genome datasets. Here we present an alternative step-by-step approach.

**Figure 1.**
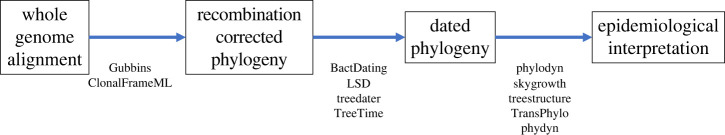
Overview of the step-by-step analytical approach. The names of some of the software tools that can be used in each step are indicated under the arrows. (Online version in colour.)

The step-by-step approach is illustrated in figure 1. In the first step, a phylogeny is constructed from a genomic alignment in a way that accounts for recombination events. In the second step, this phylogeny is dated. In the third step, the dated phylogeny is interpreted in terms of a number of epidemiological properties. Many software packages are available to perform each of these steps, including but not limited to the ones named in figure 1, although it is worth noting that many of these tools have emerged only in the past few years, and so are still work in progress and expected to improve in the near future. In this article we review each of the steps of this approach in turn. We also pay special attention to the ‘cracks' between the steps, since these are often ignored in articles that focus on each of the steps rather than the whole step-by-step approach. Finally, we demonstrate the usability of this approach by applying it to a complete collection of *Staphylococcus aureus* ST239 genomes.

## 2. Recombination-aware phylogenetic analysis

Even a relatively low amount of recombination can invalidate the results of phylogenetic tools if not accounted for [14,15]. It is therefore essential to detect recombination events to correctly reconstruct the clonal genealogy, that is the phylogenetic relationship between genomes when the ancestral lines of recipient cells rather than donor cells are followed for each ancestral recombination event. Special phylogenetic methods have been developed for this purpose, including Gubbins [16] and ClonalFrameML [17] which is based on the ClonalFrame model [18]. However, these tools are often underexploited, typically to build a recombination-corrected tree without paying attention to the recombination events and regions that have been detected.

A lot can be learnt from studying the inferred recombination events themselves. Recombination is useful to help us understand how species are being formed [19] and the population structure within species, especially when the origin of recombination events is being investigated [20]. These recombination patterns often reflect important driving evolutionary forces such as ecology [21], adaptation [22] or selective pressures [23]. For example in *Streptococcus pneumoniae*, recombination events have been shown to be driven by antibiotic usage in a localized dataset [24] and by immune pressure in a global collection of the PMEN1 lineage [25]. The latter study also represents a good example of how the temporal signal can become much clearer once recombination is correctly accounted for [25,26]. Recombination is also useful for the analysis of genome-wide associations between genotypes and phenotypes, since it separates new genetic variants from their original genomic background [27].

**Figure 2.**
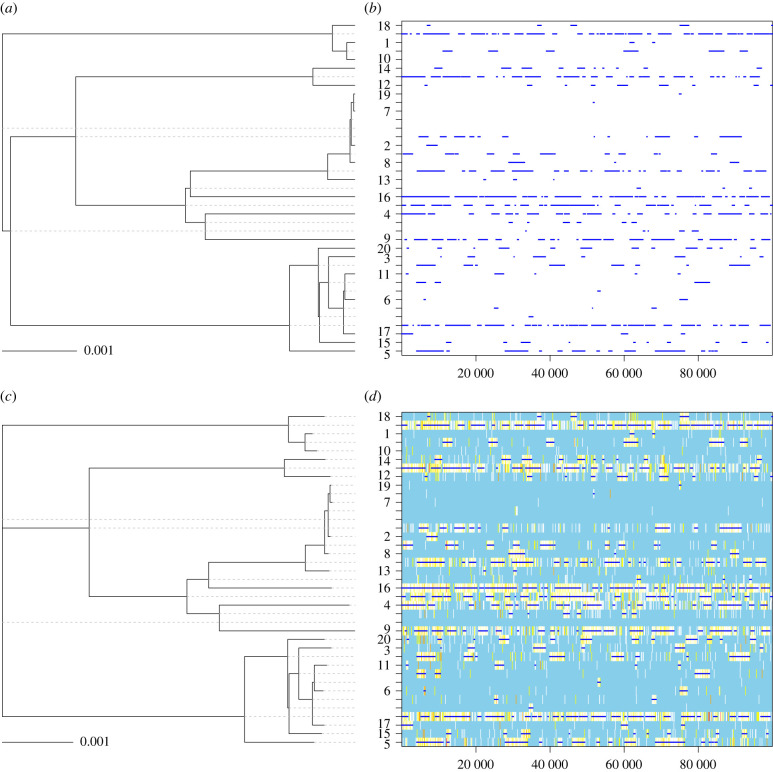
Illustration of the effect of recombination on phylogenetic inference. A phylogeny was simulated (*a*) with recombination events happening on the branches at a constant rate. (*b*) ClonalFrameML was applied to this simulated dataset, resulting in a good reconstruction of both the clonal genealogy (*c*) and recombination events (*d*).

Accounting for recombination when reconstructing phylogenies is an important starting point for many epidemiological studies. A method often used is to extract from the genomic alignment the sites that have not been affected by recombination and to build a phylogeny using these sites only. Both Gubbins and ClonalFrameML are often used in this way, to create a recombination-free alignment which is then passed on to BEAST. However, this method works only if relatively few recombination events happened throughout the tree. For example, consider the simulated dataset shown in figure 2. The true clonal genealogy is shown in figure 2*a* and the true recombination events that happened on each of the branches are shown in figure 2*b*. These data were simulated using a standard coalescent model for the phylogeny [28], a strict clock model of mutation with rate *θ*/2 = 0.005 per site, a model of recombination coming from external sources [18] with initiation rate *ρ*/2 = 0.001 per site, average length of recombination *δ* = 1500 bp and distance of the source *v* = 0.05. For clarity we used a relatively small dataset of 20 sequences of 100 000 bp each. In this simulated dataset, there was not a single site that was not affected by recombination on at least one of the branches. On the other hand, every branch had some sites unaffected by recombination (figure 2*b*).

We applied ClonalFrameML [17] to this dataset using a PhyML tree [29] as starting point. The reconstructed clonal genealogy is shown in figure 2*c* and the inferred recombination events are shown in figure 2*d*, and they are in very good agreement with the true simulated tree and events shown in figure 2*a*,*b*. ClonalFrameML correctly inferred that there was not a single site unaffected by recombination on at least one of the branches. Therefore an alignment containing only the non-recombinant sites would contain no sites, and could not be used as a starting point for further analysis. On the other hand, the inferred clonal genealogy shown in figure 2*c* can be used in our proposed step-by-step approach. It has the same topology as the true clonal genealogy (figure 2*a*) and very similar branch lengths, with a weighted Robinson-Foulds distance [30] of 0.005 between the true and ClonalFrameML trees. Gubbins [16] was also applied to this dataset using RAxML [31] as a tree builder. The correct topology was inferred, with a weighted Robinson-Foulds distance of 0.03 between the true and Gubbins trees.

## 3. Dating the ancestors in a phylogeny

Once a recombination-corrected tree has been reconstructed, it is possible to study the temporal signal in this tree and to date the common ancestors in the tree. Multiple software tools have recently been developed to perform dating on a phylogeny, including BactDating [26] which is specifically aimed at bacterial genomes, but also LSD [32], treedater [33] and TreeTime [34]. BactDating uses Bayesian statistics, whereas treedater and TreeTime are based on maximum likelihood, which is equivalent to Bayesian maximum a-posteriori (MAP) inference under a uniform prior on dates [35] so that the posterior distribution is proportional to the likelihood. It is often important to use a relaxed clock model in this step that allows the evolutionary rate to vary between lineages [5]. An additive relaxed clock model has recently been developed which is more biologically realistic and leads to better dating of pathogen phylogenies than the previous relaxed clock model [36].

In our proposed step-by-step approach, the reconstruction of a dated phylogeny and its epidemiological interpretation are separated. One disadvantage of this is that the prior (or lack of) on dates used to reconstruct the dated phylogeny is not the same as the one that would be implied by the epidemiological models used in subsequent analyses. This statistical issue could be resolved by considering the difference between the probability of a tree in the two models used for the dating and the epidemiology. For example, an importance sampler could be applied to postprocess the results, and produce corrected results that account for this difference between the two tree distributions [37]. Alternatively, the role of the prior can be assessed by comparing inference with and without the genomic data, or by comparing inference under different prior models to make sure that the posterior distributions remain consistent [38]. However, this issue is often small enough in practice to make little difference to the results [39], especially if the method used to build the dated phylogeny was based on the likelihood only, or if a mild prior was used such as the coalescent with constant population size [28].

**Figure 3.**
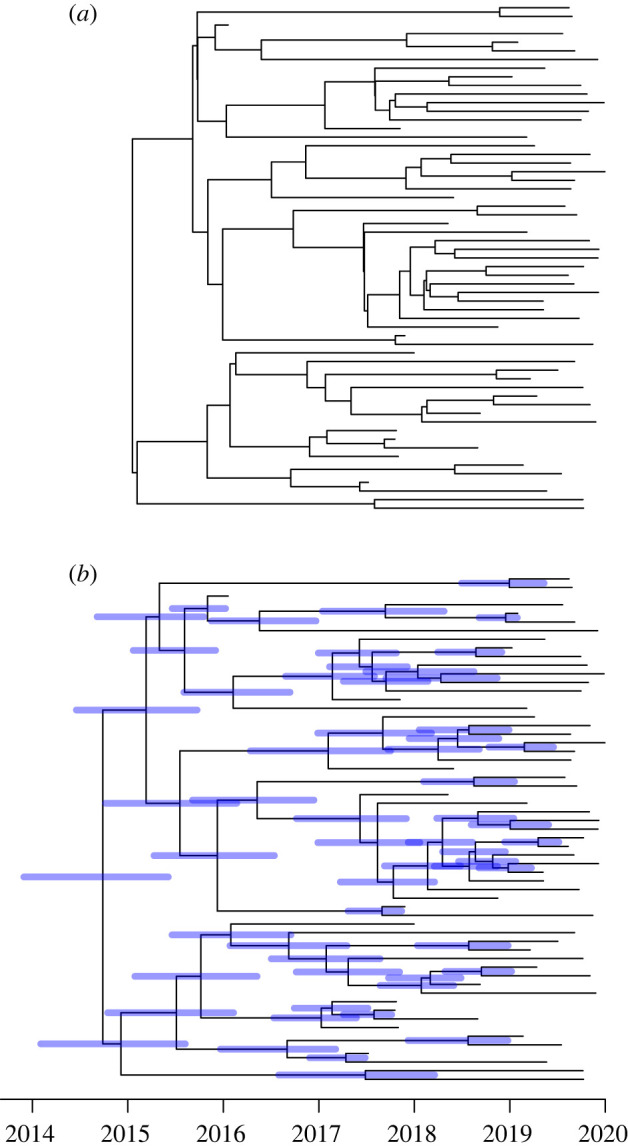
Illustration of the relative lack of effect of the prior model used for the inference of dated phylogeny. A dated phylogeny (*a*) was simulated from an epidemic model and dating was inferred (*b*) based on a coalescent model with constant population size. (Online version in colour.)

To illustrate this, we simulated five years of an outbreak model [40] with within-host diversity *N*_e_g = 0.25 year, basic reproduction number *R*_0_ = 2, generation time distribution Exponential(1) in years and sampling proportion *π* = 0.1. A total of 59 cases were sampled in this outbreak, with the samples being related to each other as shown in the ‘true’ dated phylogeny in figure 3*a*. We applied a strict clock model to this true dated phylogeny in order to produce an undated ‘observed’ phylogeny. We used a rate *μ* = 5 substitutions per year which is of the same order of magnitude as many bacterial pathogens [41]. The undated ‘observed’ phylogeny was then used, along with the known dates of sampling, to infer an ‘estimated’ dated phylogeny using BactDating [26] with prior set to the coalescent with constant population size [28]. This prior is completely different from the outbreak model that was used to generate the phylogeny [40], which is not coalescent due to the host structure and where the population size is clearly growing since the reproduction number was greater than one. Figure 3*b* shows the ‘estimated’ dated phylogeny, which is in good agreement with the ‘true’ dated phylogeny in figure 3*a* despite the complete difference between the epidemic model used for simulation and the coalescent model used for inference.

At the same time as dating is performed, the substitution rate is typically estimated which provides a useful value to compare with previous estimates [41] in order to make sure that the dating is working as expected. Statistical methods can also be used to ensure that the temporal signal is significant, in particular by using a date-randomization test [42,43]. This test involves making sure that the inferred substitution rate is larger when using the correct dates for the genomes than when the dates are permutated in at least 20 randomized datasets [42]. This method requires several runs of the dating method to be performed, and it is, therefore, useful for this to be as fast as possible, which is achieved in our step-by-step method by separating the phylogenetic inference from the dating.

Furthermore, the root of the phylogeny is typically estimated during the dating step, since the trees generated by standard phylogenetic tools are not rooted whereas dated trees are always rooted by definition, with the date of the root being the date of the last common ancestor of the whole sample. If the root has already been determined robustly, for example using one or ideally several closely related outgroups [44], then this information can be preserved during the dating. This can be achieved by using as input into the dating software a phylogeny that does not include the outgroups but that is rooted as was informed by the outgroups, and turning off the estimation of root position [26,33]. If on the other hand the root is undetermined, or arbitrarily selected for example using the midpoint method [45], then the fact that dating the phylogeny simultaneously performs rooting provides an additional reason for dating the tree, which becomes much more informative in terms of epidemiology once it is dated and rooted.

## 4. From dated phylogeny to epidemiology

A dated phylogeny is very useful to learn about the epidemiology of the bacteria under study, and sometimes the dating directly provides answers to questions of interest beyond the age of pathogens [46]. For example, several antibiotic-resistant lineages have been dated to have emerged around the time when the corresponding antibiotics were starting to be used, highlighting the link between consumption and resistance [47,48]. As another example, the dating of the common ancestors between pairs of *Clostridium difficile* patients in a hospital allowed transmission for many pairs to be ruled out, concluding that nosocomial transmission was less frequent than previously thought [49].

It can often be useful to identify clusters of significantly similar genomes in a dataset. The most commonly used approach is to use a separate dedicated algorithm that uses the genomic data for this purpose, such as HierBAPS [50], fastbaps [51] or PopPunk [52]. These methods do not make use of a phylogeny, but it is often useful to show their results overlaid on a dated phylogeny using colours for example. Another approach is to use additional non-genomic data to do the clustering given the phylogeny, as performed for example by AdaptML [53], treebreaker [54] and treeSeg [55]. Finally a third option is to try and identify directly on the dated phylogeny the lineages that seem to be ruled by different dynamics, for example using treestructure which does not rely on an explicit phylodynamic model [56] or CaveDive [57] which is focused on the detection of clonal expansions.

The dated phylogeny can also be used as a starting point for further analysis. In particular, past variations in the bacterial population size have a direct effect on the shape of the dated phylogeny, so that the population size through time can be estimated and presented as a skyline plot [58]. The methodology for performing such an analysis was originally developed within BEAST, which simultaneously estimates the dated phylogeny and the demographic function [6]. However, the step-by-step approach requires estimation of the demographic function from a dated phylogeny (figure 1), and several software tools have recently been released for this purpose including phylodyn [59,60], skygrowth [61] and mlesky [62]. Beyond a simple model of varying population size, it is also possible to fit an epidemiological compartmental model such as the susceptible-infected-recovered model [63], and therefore to estimate the parameters of this model such as the transmission rate or removal rate. Such an inference can be achieved by formulating a structured coalescent model that corresponds to the compartmental model [64,65]. Existing software for fitting such a model to a given dated phylogeny include rcolgem [66] and phydyn [67]. The same methods based on the structured coalescent can also be applied to a dated phylogeny in order to reconstruct past geographical migrations [9], although such phylogeographic inference is much more often based on discrete trait analysis, for example using the ace command from the R package ape [68] or in the NextStrain platform [69]. The worldwide spread of the current pandemic of *Vibrio cholerae* has been described using such techniques [70,71]. BEAST can also be useful in this step from dated phylogeny to epidemiology, by inputting the dated tree estimated in the previous step as the initial tree to be used in the Markov chain Monte Carlo (MCMC) and deactivating the updates on the tree. This results in a run in which the dated phylogeny is fixed, so that the run time is typically much faster. This strategy can be used for example to reconstruct a demographic function or to perform a trait analysis on large datasets [72,73].

When the genomes are densely sampled within an epidemic, it can be useful to try and reconstruct the transmission tree of who infected whom [74]. Within-host diversity and evolution is significant for many bacterial pathogens which blurs the relationships between transmission tree and phylogeny [75]. However, TransPhylo can infer the transmission tree from a dated phylogeny in a way that accounts for within-host evolution [40,76,77]. Significant uncertainty typically remains in the inferred transmission tree, which is captured by the use of Bayesian statistics within TransPhylo. More precise inference can sometimes be obtained by combining the genomic inference with epidemiological data [78].

**Figure 4.**
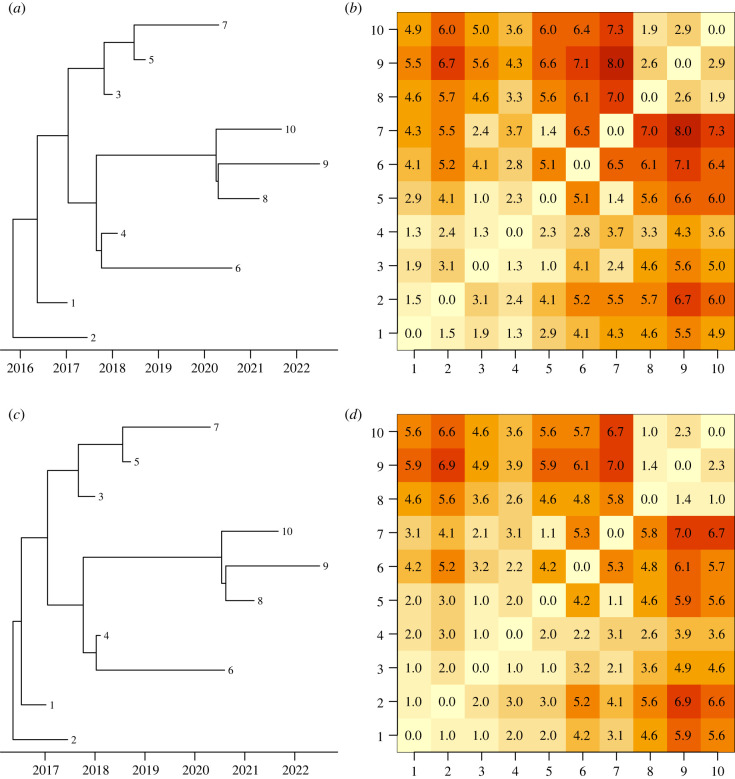
Illustration of the relative lack of effect of the uncertainty in the reconstructed dated phylogeny on interpretation as a transmission tree. Two dated phylogenies were sampled from the posterior (*a,c*) and a separate inference of the transmission tree was performed for each one (*b,d*). Values in the coloured matrices represent the distance between pairs of cases in number of transmission links. (Online version in colour.)

A drawback of separating the dating step from the interpretation step is that the uncertainty in dating is typically not passed on to the epidemiological analysis. This can be achieved by running on multiple samples from the posterior of dated phylogeny and averaging the results [79], or reweighting according to the posterior probability in the epidemiological analysis [37], but in practice the phylogenetic uncertainty is usually not accounted for. However, this is not often a significant issue in practice. To illustrate this, we simulated a dataset for a small outbreak with just 10 cases, using an epidemic model [40] with basic reproduction number *R*_0_ = 1, within-host diversity *N*_e_g = 0.25 year, mean generation time of 1 year, sampling proportion of *π* = 0.5 and a strict clock model with rate *μ* = 5 substitutions per year. The dated phylogeny was inferred using BactDating [26] and we extracted the first (after burnin) and the last trees sampled by the MCMC, as shown in figure 4*a*,*c*. We then reconstructed the transmission events using TransPhylo [40] separately for each of these two dated trees, as shown in figure 4*b*,*d*. These analyses used default parameters except that the parameters for the generation time distribution, offspring distribution, within-host diversity and sampling proportion were assumed known. In spite of small differences in the two dated phylogenies, the inferred results in terms of transmission chains were very similar.

## 5. Example of application

To illustrate the use of the step-by-step approach from bacterial genomes to epidemiology, we apply it to a state-of-the-art dataset, using only a standard laptop computer and paying particular attention to the time taken by each step. We collected all available genomes of *Staphylococcus aureus* ST239 (electronic supplementary material, table S1). This collection is made of 521 assembled genomes, only small subsets of which had been comparatively analysed in previous studies [80–83]. The genomes were collected between 1982 and 2010 from all parts of the world (451 from Asia, 46 from Europe, 18 from Americas, 2 from Africa, 2 from Oceania and 2 unknown). All genomes were aligned using MuMMER v. 3.1 [84] against the reference genome TW20 which is a member of ST239 [85] and therefore included in the collection. This resulted in a reference-anchored alignment that took only a few minutes to generate, since each pairwise alignment against the reference genome can be performed in parallel. Alternatively, assembly pipelines are often based on reference-based mapping of the sequencing reads, for example using BWA [86] and SamTools [87]. This can also be performed in parallel and results in a similar reference-anchored alignment. Note that the whole genome alignment was used as input for phylogenetic reconstruction, rather than an alignment of variant sites only, as would be produced for example using the software SNP-sites [88]. Alignments of variant sites can be used for standard phylogenetic inference if some correction is applied [89], but they cannot be used for recombination-aware phylogenetics since the genomic distance between variant sites becomes an important factor [18].

A first phylogeny was built using PhyML v. 3.3 [29] which took approximately 3 h. This was used as the starting point to build a recombination-corrected phylogeny using ClonalFrameML v. 1.12 [17], which took approximately two days to run. The same analysis using Gubbins v. 2.4.1 [16] gave very similar results and took approximately one day to run. The PhyML, ClonalFrameML and Gubbins analyses used default parameters. This step currently represents a clear bottleneck in the application of the step-by-step approach, which should be addressed in the near future through the development of new parallelised algorithms. Significant recombination was found, with a total of 198 recombination events detected throughout the phylogeny. The relative rate of recombination versus mutation was estimated to be *R*/*θ* = 0.144, meaning that on average mutation events were about f7 times more frequent than recombination events. The mean length of recombination events was estimated to be *δ* = 619 bp which is in good agreement with previous estimates for *S. aureus* [17,90,91]. The mean distance between donor and recipient was estimated to be *ν* = 0.31%, which corresponds approximately to the distance between ST239 and some of its closest relatives such as CC8 [92]. The relative effect of recombination versus mutation was therefore estimated to be r/m = *R*/*θ* **×** *ν* **×** *δ* = 0.28, so that 3 to 4 times more substitutions are caused by mutation than by recombination. These results confirm that recombination plays a role in *S. aureus* evolution, although not as dramatic as in some other bacterial pathogens [14,93,94].

**Figure 5.**
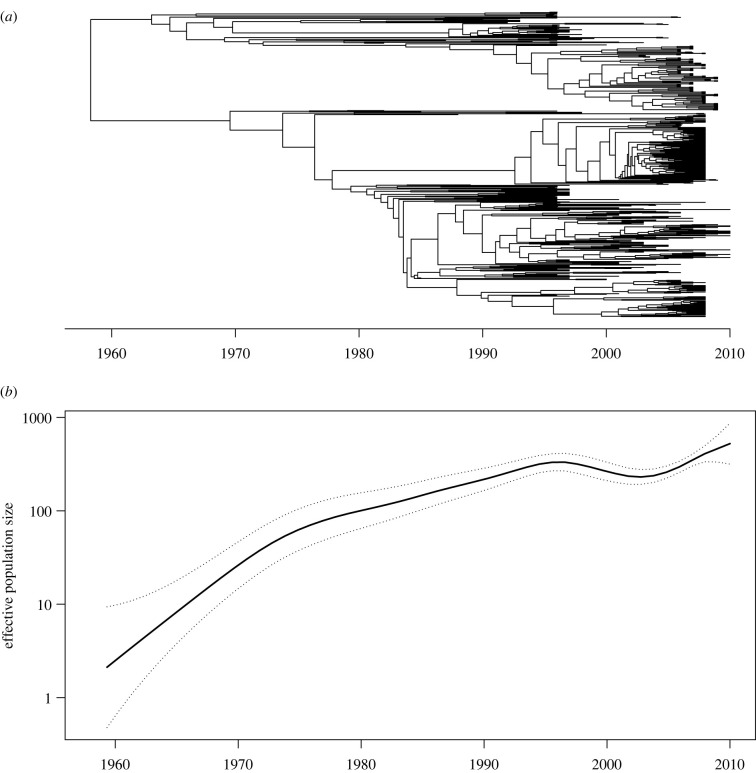
Example of application of the approach to a collection of *Staphylococcus aureus* ST239 genomes. The dated phylogeny was inferred using BactDating (*a*) and the past population size dynamics was inferred using skygrowth (*b*).

We detected a strong temporal signal in the recombination-corrected phylogeny on the basis of a regression analysis of root-to-tip distances against isolation dates (*R*^2^ = 0.57) and using the date-randomization test [42] (with 100 replicates and CR2 criterion). We, therefore, computed a dated phylogeny using BactDating v. 1.1 [26] with default parameters including use of the additive relaxed clock model [36]. This step took approximately 3 h to run for 10^6^ MCMC iterations, and the inferred dated phylogeny is shown in figure 5*a*. The isolation dates were unknown for 36 of the 521 genomes (electronic supplementary material, table S1), but BactDating can accommodate this by treating the missing dates as additional parameters that are inferred simultaneously as the dates of the common ancestors [26]. The evolutionary rate was estimated to be 7.05 substitutions per year throughout the genome, with credible interval between 6.43 and 7.67. This estimate is in good agreement with several previous estimates in ST239 [80,82] and other lineages of *S. aureus* [48]. The root of the ST239 phylogeny was estimated to have existed in 1958, with credible interval ranging between 1951 and 1965. This is again in good agreement with previous estimates and coincides with penicillins being increasingly used to treat bacterial infections [80,82,95].

We used the dated phylogeny as input into treestructure v. 0.1.2 [56] with default parameters to determine whether there were significant differences in the phylodynamic properties of sublineages within the tree. This analysis took less than a minute to perform and found no significant differences, which means that the tree can be treated as a whole in phylodynamic reconstructions [56]. We therefore applied skygrowth v. 0.3.1 [61] to the whole dated tree using the maximum a-posteriori method. This analysis took less than a minute and the estimated demographic function is shown in figure 5*b*, with an approximately exponential rise of the effective population size between 1960 and 1995, and a plateau between 1995 and 2010. This is in good agreement with previous skyline analyses of ST239 [82,95]. We do not seek to say more about the epidemiological dynamics of ST239 since our aim with this application was to test the applicability of the step-by-step method to a relatively large dataset, rather than study it in detail.

## 6. Discussion

The step-by-step approach described above (figure 1) has several drawbacks compared to an integrated approach. A practical disadvantage is that multiple tools need to be applied one after the other, with the need to make sure that the output of one tool is a suitable input for the next tool. The software tools have been developed separately, and format conversion is sometimes required when combining them, which introduces a risk of error being made. Method developers should make every effort to minimize this risk, for example by providing practical examples of source code combining new tools with pre-existing ones, and including verifications in each tool that the input is formatted as expected. Furthermore, any results from a step-by-step approach are only as good as the software components involved, many of which are imperfect and under development, which highlights the importance of thorough testing using both simulated and real datasets.

Another concern with the step-by-step approach relates to statistical soundness. In an integrated approach, a complex model is formed by combining multiple simpler models into a consistent whole, for example a model describing how the pathogen population size varied over time, another model describing how these fluctuations affect the genealogy and yet another model describing how mutation and recombination events affect the genomes given the genealogy. Inference is then performed on the combined model, with all uncertainties being accounted for simultaneously and in all directions: for example the uncertainty on a mutation event will feed into the uncertainty on the past population size, and vice versa. By contrast, in the step-by-step approach, each of the tools makes separate modelling assumptions, which may not always be consistent with each other. An example of this was discussed in §3, where the prior used for the reconstruction of a dated phylogeny was not the correct one, but figure 3 showed that the result can still be correct. Furthermore, the uncertainty can only be passed from one tool to the next in the order that they are being applied, and even in this direction it is frequent to use the best estimate from one method as the starting point of the next, without passing on any uncertainty. Again this is not necessarily a problem in practice, as illustrated in figure 4 where the uncertainty on the phylogeny had little effect on the uncertainty of the transmission tree. From a statistical point of view, the integrated approach, therefore, represents a gold standard, although statisticians have recently noted that joint inference under a combined model carries the risk that misspecification in any of the model parts can affect estimates from the others in unpredictable ways [96]. Further research is needed on this in the context of genomic epidemiology, as well as research on how to avoid the statistical issues described above with the step-by-step approach.

A key advantage to the step-by-step approach we described is that by breaking down the problem into simple steps, it becomes easier to solve, a strategy often called ‘divide and conquer’ in the computer science literature. The running time is greatly improved compared to an integrated approach, which quickly becomes intractable as more model components are combined into a large model. An example of this concerns the difficulty to integrate recombination into a phylodynamic framework [13]. A similar situation occurs when aligning sequences and building a phylogeny: in principle alignment and phylogeny would benefit from being performed simultaneously [97,98] but in practice this is too computationally challenging. The lower running time of the step-by-step approach also means that it is more scalable to the large numbers of bacterial genomes currently available, and this scalability is probably the main reason for a recent increase in popularity [39,69]. The step-by-step approach can deal with hundreds or even thousands of genomes, although some of the steps can become slow. This is particularly true for the time taken to reconstruct a recombination-corrected phylogeny, which depends not just on the number of genomes but also on their lengths, diversity, and the recombination rate. For very large datasets, it can be useful to divide them into lineages which can be analysed separately and in parallel [49,99]. New tools have recently emerged to deal with the very large numbers of sequences of SARS-CoV-2 genomes [100,101] which are likely to provide inspiration for how to improve the future scalability of bacterial genome analysis.

Perhaps even more importantly, a counterintuitive advantage of the step-by-step approach is that it is less automatic than the integrated approach. Although this may seem like a disadvantage, the fact that several software tools have to be applied one after the other brings great benefits. It allows the user to check after each step that the result makes sense before carrying out the next step. For example, if a phylogeny is clearly wrong due to contamination during sequencing, there is no point trying to apply dating of the nodes or interpreting the phylogeny in terms of epidemiology. Since each tool is focused on a simpler task, it is easier for the user to check the validity of the assumptions made, and if needed to compare models or the results of several software tools, or apply more complex models, since each step is relatively quick. These checks and refinements provide the user with a better understanding of their data and the analysis process, rather than relying on ‘black-box’ or ‘turn-key’ analysis. This is one of the most important advantages of the step-by-step approach, since it creates good conditions for a balanced interpretation of the data and results.

## Data Availability

Our paper uses data available from the NCBI website. All accession numbers are provided in electronic supplementary material, table S1 [102].

## References

[1] Loman NJ, Pallen MJ. 2015 Twenty years of bacterial genome sequencing. Nat. Rev. Microbiol. **13**, 787-794. (10.1038/nrmicro3565) 26548914

[2] Suchard MA, Lemey P, Baele G, Ayres DL, Drummond AJ, Rambaut A. 2018 Bayesian phylogenetic and phylodynamic data integration using BEAST 1.10. Virus Evol. **4**, vey016. (10.1093/ve/vey016) 29942656 PMC6007674

[3] Bouckaert R et al. 2019 BEAST 2.5: an advanced software platform for Bayesian evolutionary analysis. PLoS Comput. Biol. **15**, e1006650. (10.1371/journal.pcbi.1006650) 30958812 PMC6472827

[4] Ingle DJ, Howden BP, Duchene S. 2021 Development of phylodynamic methods for bacterial pathogens. Trends Microbiol. **29**, 788-797. (10.1016/j.tim.2021.02.008) 33736902

[5] Drummond AJ, Ho SYW, Phillips MJ, Rambaut A. 2006 Relaxed phylogenetics and dating with confidence. PLoS Biol. **4**, e88. (10.1371/journal.pbio.0040088) 16683862 PMC1395354

[6] Drummond AJ, Rambaut A, Shapiro B, Pybus OG. 2005 Bayesian coalescent inference of past population dynamics from molecular sequences. Mol. Biol. Evol. **22**, 1185-1192. (10.1093/molbev/msi103) 15703244

[7] Lemey P, Rambaut A, Drummond AJ, Suchard M. 2009 Bayesian phylogeography finds its roots. PLoS Comput. Biol. **5**, e1000520. (10.1371/journal.pcbi.1000520) 19779555 PMC2740835

[8] Lemey P, Rambaut A, Welch JJ, Suchard MA. 2010 Phylogeography takes a relaxed random walk in continuous space and time. Mol. Biol. Evol. **27**, 1877-1885. (10.1093/molbev/msq067) 20203288 PMC2915639

[9] De Maio N, Wu C-H, O'Reilly KM, Wilson D. 2015 New routes to phylogeography: a Bayesian structured coalescent approximation. PLoS Genet. **11**, e1005421. (10.1371/journal.pgen.1005421) 26267488 PMC4534465

[10] Hall M, Woolhouse M, Rambaut A. 2015 Epidemic reconstruction in a phylogenetics framework: transmission trees as partitions of the node set. PLOS Comput. Biol. **11**, e1004613. (10.1371/journal.pcbi.1004613) 26717515 PMC4701012

[11] De Maio N, Wu C-H, Wilson DJ. 2016 SCOTTI: efficient reconstruction of transmission within outbreaks with the structured coalescent. PLoS Comput. Biol. **12**, e1005130. (10.1371/journal.pcbi.1005130) 27681228 PMC5040440

[12] Didelot X, Lawson DJ, Darling AE, Falush D. 2010 Inference of homologous recombination in bacteria using whole-genome sequences. Genetics **186**, 1435-1449. (10.1534/genetics.110.120121) 20923983 PMC2998322

[13] Vaughan TG, Welch D, Drummond AJ, Biggs PJ, George T, French NP. 2017 Inferring ancestral recombination graphs from bacterial genomic data. Genetics **205**, 857-870. (10.1534/genetics.116.193425) 28007885 PMC5289856

[14] Didelot X, Maiden MCJ. 2010 Impact of recombination on bacterial evolution. Trends Microbiol. **18**, 315-322. (10.1016/j.tim.2010.04.002) 20452218 PMC3985120

[15] Hedge J, Wilson DJ. 2014 Bacterial phylogenetic reconstruction from whole genomes is robust to recombination but demographic inference is not. MBio **5**, e02158-14. (10.1128/mBio.02158-14.Editor) 25425237 PMC4251999

[16] Croucher NJ, Page AJ, Connor TR, Delaney AJ, Keane JA, Bentley SD, Parkhill J, Harris SR. 2015 Rapid phylogenetic analysis of large samples of recombinant bacterial whole genome sequences using Gubbins. Nucleic Acids Res. **43**, e15. (10.1093/nar/gku1196) 25414349 PMC4330336

[17] Didelot X, Wilson DJ. 2015 ClonalFrameML: efficient inference of recombination in whole bacterial genomes. PLoS Comput. Biol. **11**, e1004041. (10.1371/journal.pcbi.1004041) 25675341 PMC4326465

[18] Didelot X, Falush D. 2007 Inference of bacterial microevolution using multilocus sequence data. Genetics **175**, 1251-1266. (10.1534/genetics.106.063305) 17151252 PMC1840087

[19] Krause DJ, Whitaker RJ. 2015 Inferring speciation processes from patterns of natural variation in microbial genomes. Syst. Biol. **64**, 926-935. (10.1093/sysbio/syv050) 26316424 PMC4604833

[20] Didelot X et al. 2011 Recombination and population structure in *Salmonella enterica*. PLoS Genet. **7**, e1002191. (10.1371/journal.pgen.1002191) 21829375 PMC3145606

[21] Sheppard SK et al. 2013 Progressive genome-wide introgression in agricultural *Campylobacter coli*. Mol. Ecol. **22**, 1051-1064. (10.1111/mec.12162) 23279096 PMC3749442

[22] Sheppard SK et al. 2013 Genome-wide association study identifies vitamin B5 biosynthesis as a host specificity factor in *Campylobacter*. Proc. Natl Acad. Sci. USA **110**, 11 923-11 927. (10.5061/dryad.28n35) PMC3718156 23818615

[23] Hedge J, Wilson DJ. 2016 Practical approaches for detecting selection in microbial genomes. PLoS Comput. Biol. **12**, e1004739. (10.1371/journal.pcbi.1004739) 26867134 PMC4750996

[24] Chewapreecha C et al. 2014 Dense genomic sampling identifies highways of pneumococcal recombination. Nat. Genet. **46**, 305-309. (10.1038/ng.2895) 24509479 PMC3970364

[25] Croucher NJ et al. 2011 Rapid pneumococcal evolution in response to clinical interventions. Science **331**, 430-434. (10.1126/science.1198545) 21273480 PMC3648787

[26] Didelot X, Croucher NJ, Bentley SD, Harris SR, Wilson DJ. 2018 Bayesian inference of ancestral dates on bacterial phylogenetic trees. Nucleic Acids Res. **46**, e134. (10.1093/nar/gky783) 30184106 PMC6294524

[27] Collins C, Didelot X. 2018 A phylogenetic method to perform genome-wide association studies in microbes that accounts for population structure and recombination. PLoS Comput. Biol. **14**, e1005958. (10.1371/journal.pcbi.1005958) 29401456 PMC5814097

[28] Kingman JFC. 1982 The coalescent. Stoch. Process. Appl. **13**, 235-248. (10.1016/0304-4149(82)90011-4)

[29] Guindon S, Dufayard J-F, Lefort V, Anisimova M, Hordijk W, Gascuel O. 2010 New algorithms and methods to estimate maximum-likelihood phylogenies: assessing the performance of PhyML 3.0. Syst. Biol. **59**, 307-321. (10.1093/sysbio/syq010) 20525638

[30] Robinson DF, Foulds LR. 1981 Comparison of phylogenetic trees. Math. Biosci. **53**, 131-147. (10.1016/0025-5564(81)90043-2)

[31] Stamatakis A. 2014 RAxML version 8: a tool for phylogenetic analysis and post-analysis of large phylogenies. Bioinformatics **30**, 1312-1313. (10.1093/bioinformatics/btu033) 24451623 PMC3998144

[32] To T-H, Jung M, Lycett S, Gascuel O. 2016 Fast dating using least-squares criteria and algorithms. Syst. Biol. **65**, 82-97. (10.1093/sysbio/syv068) 26424727 PMC4678253

[33] Volz EM, Frost SDW. 2017 Scalable relaxed clock phylogenetic dating. Virus Evol. **3**, vex025. (10.1093/ve/vex025)

[34] Sagulenko P, Puller V, Neher RA. 2018 TreeTime: maximum likelihood phylodynamic analysis. Virus Evol. **4**, vex042. (10.1101/153494) 29340210 PMC5758920

[35] Guindon S. 2010 Bayesian estimation of divergence times from large sequence alignments. Mol. Biol. Evol. **27**, 1768-1781. (10.1093/molbev/msq060) 20194424

[36] Didelot X, Siveroni I, Volz EM. 2021 Additive uncorrelated relaxed clock models for the dating of genomic epidemiology phylogenies. Mol. Biol. Evol. **38**, 307-317. (10.1093/molbev/msaa193) 32722797 PMC8480190

[37] Meligkotsidou L, Fearnhead P. 2007 Postprocessing of genealogical trees. Genetics **177**, 347-358. (10.1534/genetics.107.071910) 17565950 PMC2013683

[38] Boskova V, Stadler T, Magnus C. 2018 The influence of phylodynamic model specifications on parameter estimates of the Zika virus epidemic. Virus Evol. **4**, 1-14. (10.1093/ve/vex044) PMC5789282 29403651

[39] Duchene S, Duchene DA, Geoghegan JL, Dyson ZA, Hawkey J, Holt KE. 2018 Inferring demographic parameters in bacterial genomic data using Bayesian and hybrid phylogenetic methods. BMC Evol. Biol. **18**, 1-11. (10.1186/s12862-018-1210-5) 29914372 PMC6006949

[40] Didelot X, Fraser C, Gardy J, Colijn C. 2017 Genomic infectious disease epidemiology in partially sampled and ongoing outbreaks. Mol. Biol. Evol. **34**, 997-1007. (10.1093/molbev/msw275) 28100788 PMC5850352

[41] Duchêne S, Holt KE, Weill F-X, Le Hello S, Hawkey J, Edwards DJ, Fourment M, Holmes EC. 2016 Genome-scale rates of evolutionary change in bacteria. Microb. Genomics **2**, e000094. (10.1101/069492) PMC5320706 28348834

[42] Duchêne S, Duchêne D, Holmes EC, Ho SYW. 2015 The performance of the date-randomization test in phylogenetic analyses of time-structured virus data. Mol. Biol. Evol. **32**, 1895-1906. (10.1093/molbev/msv056) 25771196

[43] Duchêne S, Geoghegan JL, Holmes EC, Ho SYW. 2016 Estimating evolutionary rates using time-structured data: a general comparison of phylogenetic methods. Bioinformatics **32**, 3375-3379. (10.1093/bioinformatics/btw421) 27412094

[44] Tarrío R, Rodríguez-Trelles F, Ayala FJ. 2000 Tree rooting with outgroups when they differ in their nucleotide composition from the ingroup: the *Drosophila saltans* and *willistoni* groups, a case study. Mol. Phylogenet. Evol. **16**, 344-349. (10.1006/mpev.2000.0813) 10991788

[45] Hess PN, De Moraes Russo CA. 2007 An empirical test of the midpoint rooting method. Biol. J. Linn. Soc. **92**, 669-674. (10.1111/j.1095-8312.2007.00864.x) PMC7110036 32287391

[46] Achtman M. 2016 How old are bacterial pathogens? Proc. R. Soc. B **283**, 20160990. (10.1098/rspb.2016.0990) PMC5013766 27534956

[47] Ward MJ, Gibbons CL, McAdam PR, van Bunnik BAD, Girvan EK, Edwards GF, Fitzgerald JR, Woolhouse MEJ. 2014 Time-scaled evolutionary analysis of the transmission and antibiotic resistance dynamics of *Staphylococcus aureus* clonal complex 398. Appl. Environ. Microbiol. **80**, 7275-7282. (10.1128/AEM.01777-14) 25239891 PMC4249192

[48] Holden MTG et al. 2013 A genomic portrait of the emergence, evolution and global spread of a methicillin resistant *Staphylococcus aureus* pandemic. Genome Res. **23**, 653-664. (10.1101/gr.147710.112) 23299977 PMC3613582

[49] Didelot X et al. 2012 Microevolutionary analysis of *Clostridium difficile* genomes to investigate transmission. Genome Biol. **13**, R118. (10.1186/gb-2012-13-12-r118) 23259504 PMC4056369

[50] Cheng L, Connor TR, Sirén J, Aanensen DM, Corander J. 2013 Hierarchical and spatially explicit clustering of DNA sequences with BAPS software. Mol. Biol. Evol. **30**, 1224-1228. (10.1093/molbev/mst028) 23408797 PMC3670731

[51] Tonkin-Hill G, Lees JA, Bentley SD, Frost SDW, Corander J. 2019 Fast hierarchical Bayesian analysis of population structure. Nucleic Acids Res. **47**, 5539-5549. (10.1093/nar/gkz361) 31076776 PMC6582336

[52] Lees JA, Harris SR, Tonkin-Hill G, Gladstone RA, Lo SW, Weiser JN, Corander J, Bentley SD, Croucher NJ. 2019 Fast and flexible bacterial genomic epidemiology with PopPUNK. Genome Res. **29**, 304-316. (10.1101/gr.241455.118) 30679308 PMC6360808

[53] Hunt DEDE, David LA, Gevers D, Preheim SP, Alm EJ, Polz MF. 2008 Resource partitioning and sympatric differentiation among closely related bacterioplankton. Science **1081**, 1081. (10.1126/science.1157890) 18497299

[54] Ansari MA, Didelot X. 2016 Bayesian inference of the evolution of a phenotype distribution on a phylogenetic tree. Genetics **204**, 89-98. (10.1534/genetics.116.190496) 27412711 PMC5012407

[55] Behr M, Ansari MA, Munk A, Holmes C. 2019 Testing for dependence on tree structures. bioRxiv, 622811. (10.1101/622811) PMC7211961 32321827

[56] Volz EM, Wiuf C, Grad YH, Frost SDW, Dennis AM, Didelot X. 2020 Identification of hidden population structure in time-scaled phylogenies. Syst. Biol. **69**, 884-896. (10.1093/sysbio/syaa009) 32049340 PMC8559910

[57] Helekal D, Ledda A, Volz E, Wyllie D, Didelot X. 2021 Bayesian inference of clonal expansions in a dated phylogeny. Syst. Biol. syab095. (10.1093/sysbio/syab095) PMC9366454 34893904

[58] Ho SYW, Shapiro B. 2011 Skyline-plot methods for estimating demographic history from nucleotide sequences. Mol. Ecol. Resour. **11**, 423-434. (10.1111/j.1755-0998.2011.02988.x) 21481200

[59] Lan S, Palacios JA, Karcher M, Minin VN, Shahbaba B. 2015 An efficient Bayesian inference framework for coalescent-based nonparametric phylodynamics. Bioinformatics **31**, 3282-3289. (10.1093/bioinformatics/btv378) 26093147 PMC4795633

[60] Karcher MD, Palacios JA, Lan S, Minin VN. 2017 phylodyn: an R package for phylodynamic simulation and inference. Mol. Ecol. Resour. **17**, 96-100. (10.1111/1755-0998.12630) 27801980 PMC5466693

[61] Volz EM, Didelot X. 2018 Modeling the growth and decline of pathogen effective population size provides insight into epidemic dynamics and drivers of antimicrobial resistance. Syst. Biol. **67**, 719-728. (10.1093/sysbio/syy007) 29432602 PMC6005154

[62] Didelot X, Geidelberg L, Volz E. 2021 Model design for non-parametric phylodynamic inference and applications to pathogen surveillance. *bioRxiv*, 427056. (10.1101/2021.01.18.427056)

[63] Tang L, Zhou Y, Wang L, Purkayastha S, Zhang L, He J, Wang F, Song PXK. 2020 A review of multi-compartment infectious disease models. Int. Stat. Rev. **88**, 462-513. (10.1111/insr.12402) 32834402 PMC7436714

[64] Volz EM. 2012 Complex population dynamics and the coalescent under neutrality. Genetics **190**, 187-201. (10.1534/genetics.111.134627) 22042576 PMC3249372

[65] Volz EM, Koelle K, Bedford T. 2013 Viral phylodynamics. PLoS Comput. Biol. **9**, e1002947. (10.1371/journal.pcbi.1002947) 23555203 PMC3605911

[66] Volz EM, Kosakovsky Pond SL, Ward MJ, Leigh Brown AJ, Frost SDW. 2009 Phylodynamics of infectious disease epidemics. Genetics **183**, 1421-1430. (10.1534/genetics.109.106021) 19797047 PMC2787429

[67] Volz EM, Siveroni I. 2018 Bayesian phylodynamic inference with complex models. PLoS Comput. Biol. **14**, e1006546. (10.1371/journal.pcbi.1006546) 30422979 PMC6258546

[68] Paradis E, Schliep K. 2019 Ape 5.0: an environment for modern phylogenetics and evolutionary analyses in R. Bioinformatics **35**, 526-528. (10.1093/bioinformatics/bty633) 30016406

[69] Hadfield J, Megill C, Bell SM, Huddleston J, Potter B, Callender C, Sagulenko P, Bedford T, Neher RA. 2018 NextStrain: real-time tracking of pathogen evolution. Bioinformatics **34**, 4121-4123. (10.1093/bioinformatics/bty407) 29790939 PMC6247931

[70] Mutreja A et al. 2011 Evidence for several waves of global transmission in the seventh cholera pandemic. Nature **477**, 462-465. (10.1038/nature10392) 21866102 PMC3736323

[71] Didelot X, Pang B, Zhou Z, McCann A, Ni P, Li D, Achtman M, Kan B. 2015 The role of China in the global spread of the current cholera pandemic. PLoS Genet. **11**, e1005072. (10.1371/journal.pgen.1005072) 25768799 PMC4358972

[72] Dellicour S et al. 2021 A phylodynamic workflow to rapidly gain insights into the dispersal history and dynamics of SARS-CoV-2 lineages. Mol. Biol. Evol. **38**, 1608-1613. (10.1093/molbev/msaa284) 33316043 PMC7665608

[73] Duchene S, Featherstone L, De Blasio BF, Holmes EC, Bohlin J, Pettersson JHO. 2021 The impact of public health interventions in the Nordic countries during the first year of SARS-CoV-2 transmission and evolution. Eurosurveillance **26**, 2001996. (10.2807/1560-7917.ES.2021.26.44.2001996) PMC8569925 34738512

[74] Jombart T, Eggo RM, Dodd PJ, Balloux F. 2011 Reconstructing disease outbreaks from genetic data: a graph approach. Heredity **106**, 383-390. (10.1038/hdy.2010.78) 20551981 PMC3183872

[75] Didelot X, Walker AS, Peto TE, Crook DW, Wilson DJ. 2016 Within-host evolution of bacterial pathogens. Nat. Rev. Microbiol. **14**, 150-162. (10.1038/nrmicro.2015.13) 26806595 PMC5053366

[76] Didelot X, Gardy J, Colijn C. 2014 Bayesian inference of infectious disease transmission from whole genome sequence data. Mol. Biol. Evol. **31**, 1869-1879. (10.1093/molbev/msu121) 24714079 PMC4069612

[77] Didelot X, Kendall M, Xu Y, White PJ, McCarthy N. 2021 Genomic epidemiology analysis of infectious disease outbreaks using TransPhylo. Curr. Protoc. **1**, 1-23. (10.1002/cpz1.60) PMC7995038 33617114

[78] Biek R, Pybus OG, Lloyd-Smith JO, Didelot X. 2015 Measurably evolving pathogens in the genomic era. Trends Ecol. Evol. **30**, 306-313. (10.1016/j.tree.2015.03.009) 25887947 PMC4457702

[79] Nylander JAA, Olsson U, Alström P, Sanmartín I. 2008 Accounting for phylogenetic uncertainty in biogeography: a Bayesian approach to dispersal-vicariance analysis of the thrushes (Aves: Turdus). Syst. Biol. **57**, 257-268. (10.1080/10635150802044003) 18425716

[80] Harris SRR et al. 2010 Evolution of MRSA during hospital transmission and intercontinental spread. Science **327**, 469-474. (10.1126/science.1182395) 20093474 PMC2821690

[81] Castillo-Ramírez S et al. 2012 Phylogeographic variation in recombination rates within a global clone of methicillin-resistant *Staphylococcus aureus*. Genome Biol. **13**, R126. (10.1186/gb-2012-13-12-r126) 23270620 PMC3803117

[82] Hsu LY et al. 2015 Evolutionary dynamics of methicillin-resistant *Staphylococcus aureus* within a healthcare system. Genome Biol. **16**, 1-13. (10.1186/s13059-015-0643-z) 25903077 PMC4407387

[83] Tong SYC et al. 2015 Genome sequencing defines phylogeny and spread of methicillin-resistant *Staphylococcus aureus* in a high transmission setting. Genome Res. **25**, 111-118. (10.1101/gr.174730.114.Freely) 25491771 PMC4317166

[84] Kurtz S, Phillippy A, Delcher AL, Smoot M, Shumway M, Antonescu C, Salzberg SL. 2004 Versatile and open software for comparing large genomes. Genome Biol. **5**, R12. (10.1186/gb-2004-5-2-r12) 14759262 PMC395750

[85] Holden MTG et al. 2010 Genome sequence of a recently emerged, highly transmissible, multi-antibiotic- and antiseptic-resistant variant of methicillin-resistant *Staphylococcus aureus*, sequence type 239 (TW). J. Bacteriol. **192**, 888-892. (10.1128/JB.01255-09) 19948800 PMC2812470

[86] Li H, Durbin R. 2009 Fast and accurate short read alignment with Burrows–Wheeler transform. Bioinformatics **25**, 1754-1760. (10.1093/bioinformatics/btp324) 19451168 PMC2705234

[87] Li H, Handsaker B, Wysoker A, Fennell T, Ruan J, Homer N, Marth G, Abecasis G, Durbin R. 2009 The Sequence Alignment/Map (SAM) format and SAMtools. Bioinformatics **25**, 2078-2079. (10.1093/bioinformatics/btp352) 19505943 PMC2723002

[88] Page AJ, Taylor B, Delaney AJ, Soares J, Seemann T, Keane JA, Harris SR. 2016 SNP-sites: rapid efficient extraction of SNPs from multi-FASTA alignments. Microb. Genom. **2**, e000056. (10.1099/mgen.0.000056) 28348851 PMC5320690

[89] Lewis PO. 2001 A likelihood approach to estimating phylogeny from discrete morphological character data. Syst. Biol. **50**, 913-925. (10.1080/106351501753462876) 12116640

[90] Méric G et al. 2015 Ecological overlap and horizontal gene transfer in *Staphylococcus aureus* and *Staphylococcus epidermidis*. Genome Biol. Evol. **7**, 1313-1328. (10.1093/gbe/evv066) 25888688 PMC4453061

[91] Everitt RG et al. 2014 Mobile elements drive recombination hotspots in the core genome of *Staphylococcus aureus*. Nat. Commun. **5**, 3956. (10.1038/ncomms4956) 24853639 PMC4036114

[92] Richardson EJ et al. 2018 Gene exchange drives the ecological success of a multi-host bacterial pathogen. Nat. Ecol. Evol. **2**, 1468-1478. (10.1038/s41559-018-0617-0) 30038246 PMC7610605

[93] Vos M, Didelot X. 2009 A comparison of homologous recombination rates in bacteria and archaea. ISME J. **3**, 199-208. (10.1038/ismej.2008.93) 18830278

[94] Yahara K, Didelot X, Jolley KA, Kobayashi I, Maiden MCJ, Sheppard SK, Falush D. 2016 The landscape of realized homologous recombination in pathogenic bacteria. Mol. Biol. Evol. **33**, 456-471. (10.1093/molbev/msv237) 26516092 PMC4866539

[95] Baines SL et al. 2015 Convergent adaptation in the dominant global hospital clone ST239 of methicillin-resistant *Staphylococcus aureus*. MBio **6**, e00080. (10.1128/mBio.00080-15) 25736880 PMC4358018

[96] Jacob PE, Murray LM, Holmes CC, Robert CP. 2017 Better together? Statistical learning in models made of modules. *arXiv.* (10.48550/ARXIV.1708.08719)

[97] Novák Á, Miklós I, Lyngsø R, Hein J. 2008 StatAlign: an extendable software package for joint Bayesian estimation of alignments and evolutionary trees. Bioinformatics **24**, 2403-2404. (10.1093/bioinformatics/btn457) 18753153

[98] Herman JL, Challis CJ, Novák Á, Hein J, Schmidler SC. 2014 Simultaneous Bayesian estimation of alignment and phylogeny under a joint model of protein sequence and structure. Mol. Biol. Evol. **31**, 2251-2266. (10.1093/molbev/msu184) 24899668 PMC4137710

[99] Ektefaie Y, Dixit A, Freschi L, Farhat MR. 2021 Globally diverse *Mycobacterium tuberculosis* resistance acquisition: a retrospective geographical and temporal analysis of whole genome sequences. Lancet Microbe **2**, e96-e104. (10.1016/S2666-5247(20)30195-6) 33912853 PMC8078851

[100] Ferreira R-C et al. 2021 CoVizu: rapid analysis and visualization of the global diversity of SARS-CoV-2 genomes. Virus Evol. **7**, 1-7. (10.1101/2021.07.20.453079) PMC10131274 37124703

[101] Turakhia Y, Thornlow B, Hinrichs AS, De Maio N, Gozashti L, Lanfear R, Haussler D, Corbett-Detig R. 2021 Ultrafast Sample placement on Existing tRees (UShER) enables real-time phylogenetics for the SARS-CoV-2 pandemic. Nat. Genet. **53**, 809-816. (10.1038/s41588-021-00862-7) 33972780 PMC9248294

[102] Didelot X, Parkhill J. 2022 A scalable analytical approach from bacterial genomes to epidemiology. *Figshare*. **11**, 423-434. (10.6084/m9.figshare.c.6080816) PMC9393561 35989600

